# Supplementary material for: SoxB1-mediated chromatin remodeling promotes sensory neuron differentiation in planarians
Source: Genetics. 2026 Jan 8;232(3):iyag002. doi: 10.1093/genetics/iyag002 (PMC13017442; doi:10.1093/genetics/iyag002)
Supplement: iyag002_Supplementary_Data [file iyag002_supplementary_data.zip › Supplemental_Material_Legends_GENETICS-2025-308887.docx]

**Supplemental Figure Legends**

**Supplemental Figure 1. Schematic of the assay used to assess chromatin accessibility and transcriptional changes in *gfp* control and *soxB1-2* RNAi conditions.**

**Supplemental Figure 2. Fragment size distribution and Pearson correlation analysis of ATAC-seq replicates.** (A) Fragment size distributions from each ATAC-seq replicate. Forward and reverse reads are shown in red and blue, respectively. (B) Biological replicates within control groups are highly correlated (r = 0.96; n = 2). (C) Biological replicates within *soxB1-2* RNAi groups are highly correlated (r = 0.96; n = 2).

**Supplemental Figure 3. ATAC-seq analysis reveals differences in chromatin accessibility following *soxB1-2* knockdown**. Distribution of ATAC-seq peaks across annotated genomic regions of the *S. mediterranea* genome, comparing all *gfp* control and *soxB1-2* RNAi replicates. "DA peaks" indicate regions with detectable accessibility in *gfp* controls that are absent following *soxB1-2* knockdown.

**Supplemental Figure 4. Motif enrichment analysis of differential ATAC-seq footprints using BINDetect.** (A) Raw score distributions for transcription factor binding site footprints in control and *soxB1-2* RNAi samples, showing the density of unscaled footprint scores across conditions. (B) Normalized score distributions after within-condition scaling, demonstrating comparable score distributions suitable for differential binding analysis. (C) Volcano plot of differential transcription factor binding generated by BINDetect. Positive differential binding scores indicate motifs with higher footprint occupancy in *soxB1-2* RNAi samples, whereas negative scores indicate motifs more enriched in control samples. Notably, Sox family motifs (SoxB1, SoxB2, SoxC, SoxF) and several T-box factors (e.g., Tbx2/3, Brachyury) show strong differential binding.

**Supplemental Figure 5. Heatmap of isolated differentially expressed genes (FC ≥ 1.4, p < 0.05) in *gfp* control vs. *soxB1-2* RNAi groups.** Z-scores indicate upregulation (2), no change (0), and downregulation (-2). Labeled genes represent putative sensory neural genes selected for validation via in situ hybridization.

**Supplemental Figure 6. Co-expression of *soxB1-2* and select genes identified by RNA-seq.** Single-cell expression profiles of cells co-expressing *soxB1-2* and representative RNA-seq–identified genes that are significantly downregulated following *soxB1-2* RNAi. Cell type abbreviations: sNB, sigma neoblast; zNB, zeta neoblast; gNB, gamma neoblast; EEP, early epidermal progenitor; LEP, late epidermal progenitor; E1, epidermis 1; E2, epidermis 2; Gut, intestine; PN, protonephridia; PP, parapharyngeal; Mu, muscle; N, neural; N-C, neural-ciliated.

**Supplemental Figure 7. Gene Ontology (GO) term enrichment of genes differentially expressed following soxB1-2 RNAi.** (A) GO terms associated with 134 genes shared between RNA-seq datasets from whole-worm and head-only samples. (B) GO term enrichment of 148 downregulated genes uniquely identified in whole-worm RNA-seq datasets (Ross *et al.* 2018). (C) GO term enrichment of 416 upregulated genes in RNA-seq datasets of head-only samples.

**Supplemental Figure 8. Expression patterns of genes with reduced chromatin accessibility or transcription following *soxB1-2* RNAi.** Decreased or absent expression of genes identified through ATAC-seq or RNA-seq as downregulated following *soxB1-2* RNAi. *dd_11972* shows reduced expression in the head tip and across the epidermis, while *dd_7770*, *dd_45275*, *dnai1*, and s*lc25a-21* exhibit complete loss of expression. *pkd1L-2* serves as a positive control for confirming the knockdown of *soxB1-2* downstream targets via in situ hybridization. Sample size: For controls, N ≥ 3 worms were analyzed, and all samples showed consistent expression patterns. For RNAi-treated animals, the number of biological replicates (N) is indicated at the top left of each treatment group.

**Supplemental Figure 9. Experimental design for *mecom* RNA-seq and heatmap of candidate targets meeting differential expression cut-offs.** (A) Schematic of the assay used to assess differential gene expression in *gfp* control and *mecom* RNAi conditions. (B) Validation of *mecom* transcript loss at the time point used for RNA-seq sample collection. For controls, N ≥ 3 worms were analyzed, and all samples showed consistent expression patterns. For RNAi-treated animals, the number of biological replicates (N) is indicated at the top left of the treatment group. (C) Heatmap of differentially expressed genes (FC ≥ 1.4, p < 0.1) in *gfp* control vs. *mecom* RNAi groups. Z-scores indicate upregulation (2), no change (0), and downregulation (-2). Labeled genes are putative mechanosensory genes identified in RNA-seq experiments performed on *mecom* RNAi samples.

**Supplemental Figure 10. Experimental design for *castor* RNA-seq and heatmap of candidate targets meeting differential expression cut-offs.** (A) Schematic of the assay used to assess differential gene expression in *gfp* control and *castor* RNAi conditions. (B) Validation of *castor* transcript loss at the time point used for RNA-seq sample collection. For controls, N ≥ 3 worms were analyzed, and all samples showed consistent expression patterns. For RNAi-treated animals, the number of biological replicates (N) is indicated at the top left of the treatment group. (C) Heatmap of differentially expressed genes (FC ≥ 1.4, p < 0.1) in *gfp* control vs. *castor* RNAi groups. Z-scores indicate upregulation (2), no change (0), and downregulation (-2). Labeled genes are putative mechanosensory genes identified in RNA-seq experiments performed on *castor* RNAi samples.

**Supplemental Table Files**

**Supplemental Table 1.** List of ATAC-seq peaks detected in control and RNAi groups, including motif enrichment analysis results.

**Supplemental Table 2.** Differentially expressed genes in soxB1-2 RNAi versus control groups and comparison of results between head-only and whole-body RNA-seq datasets.

**Supplemental Table 3.** Genes overlapping between ATAC-seq and RNA-seq datasets and associated single-cell cluster enrichment analysis.

**Supplemental Table 4.** DESeq2 differential expression analysis results for *Smed-mecom* RNAi.

**Supplemental Table 5.** DESeq2 differential expression analysis results for *Smed-castor* RNAi.
