# Supplementary figures and images for: SoxB1-mediated chromatin remodeling promotes sensory neuron differentiation in planarians
Source: Genetics. 2026 Jan 8;232(3):iyag002. doi: 10.1093/genetics/iyag002 (PMC13017442; doi:10.1093/genetics/iyag002)

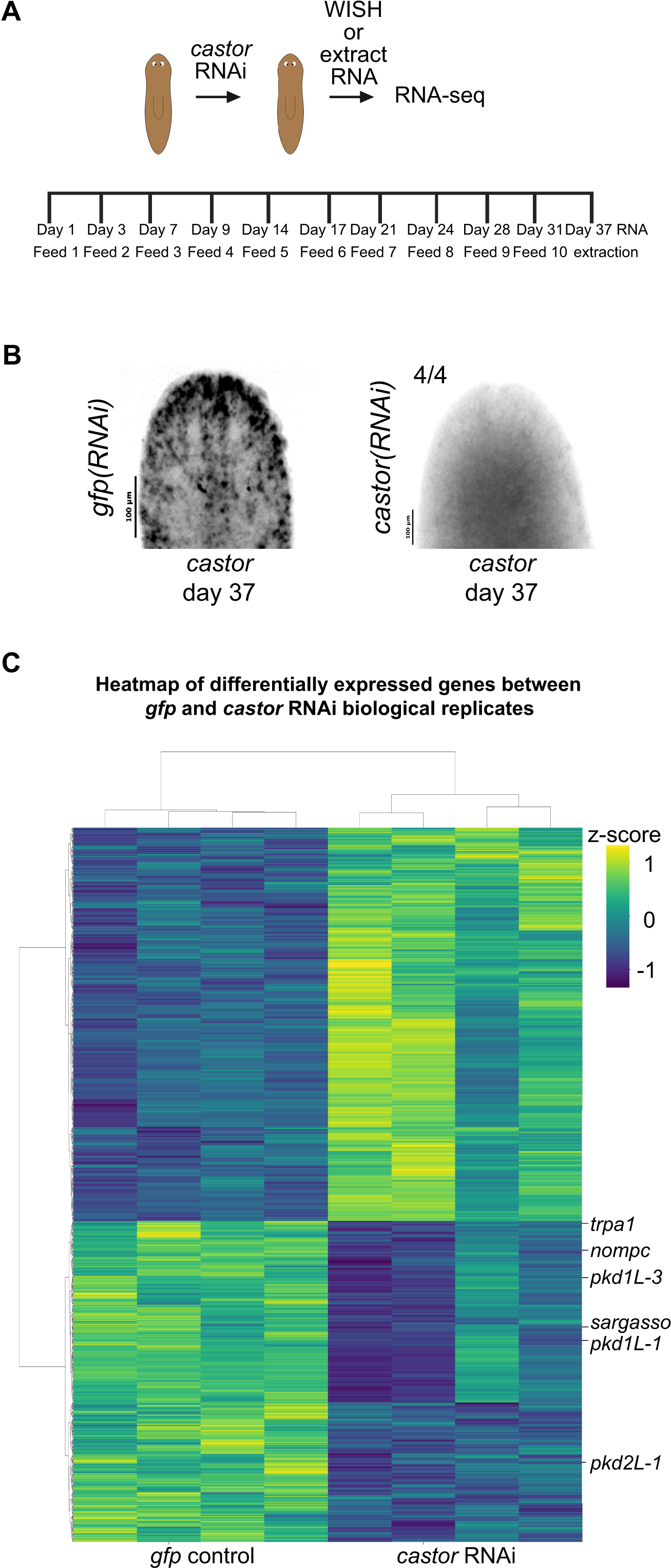

Supplement: iyag002_Supplementary_Data [file iyag002_supplementary_data.zip › Supplemental_Figure_10_GENETICS-2025-308887.tif]

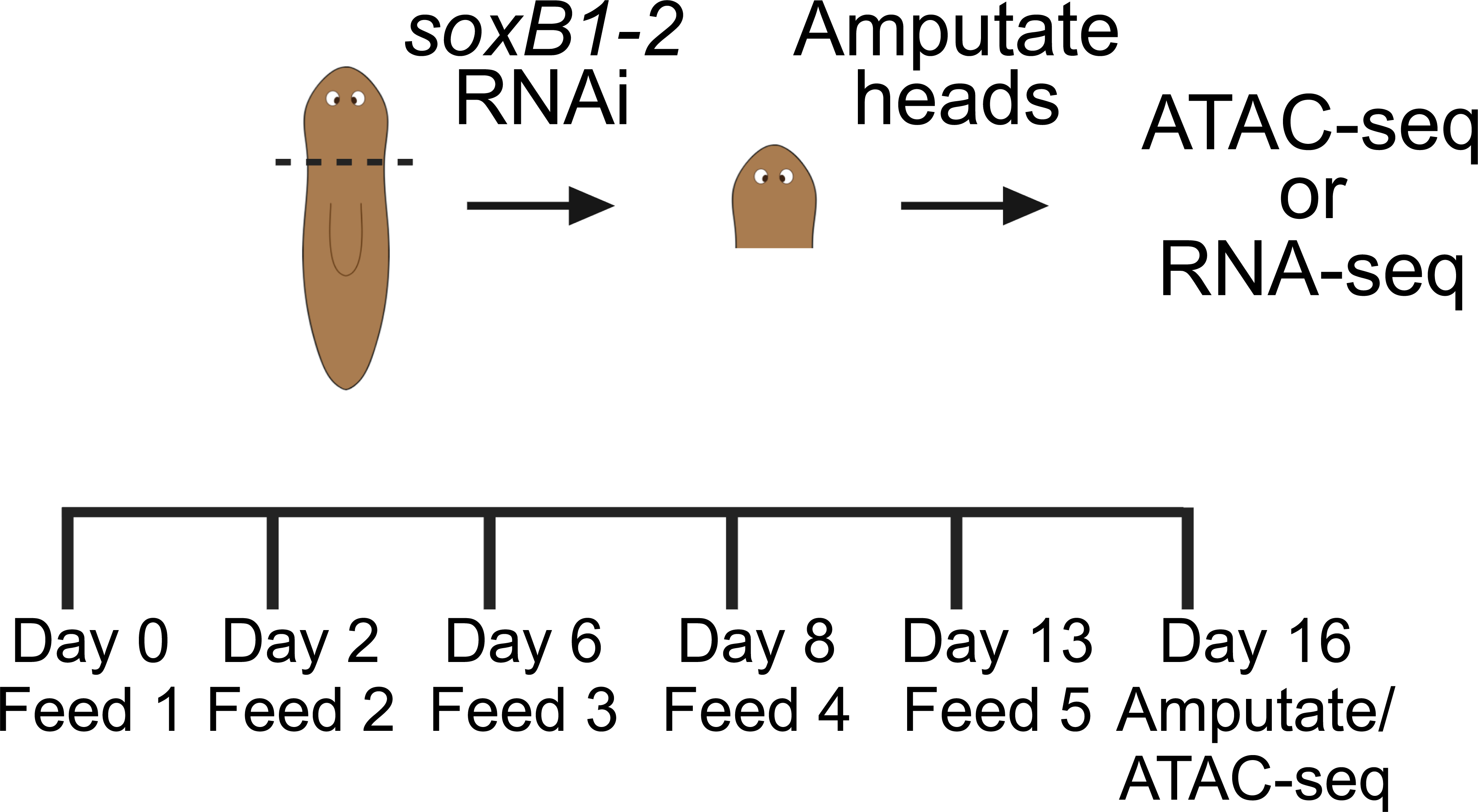

Supplement: iyag002_Supplementary_Data [file iyag002_supplementary_data.zip › Supplemental_Figure_1_GENETICS-2025-308887.tif]

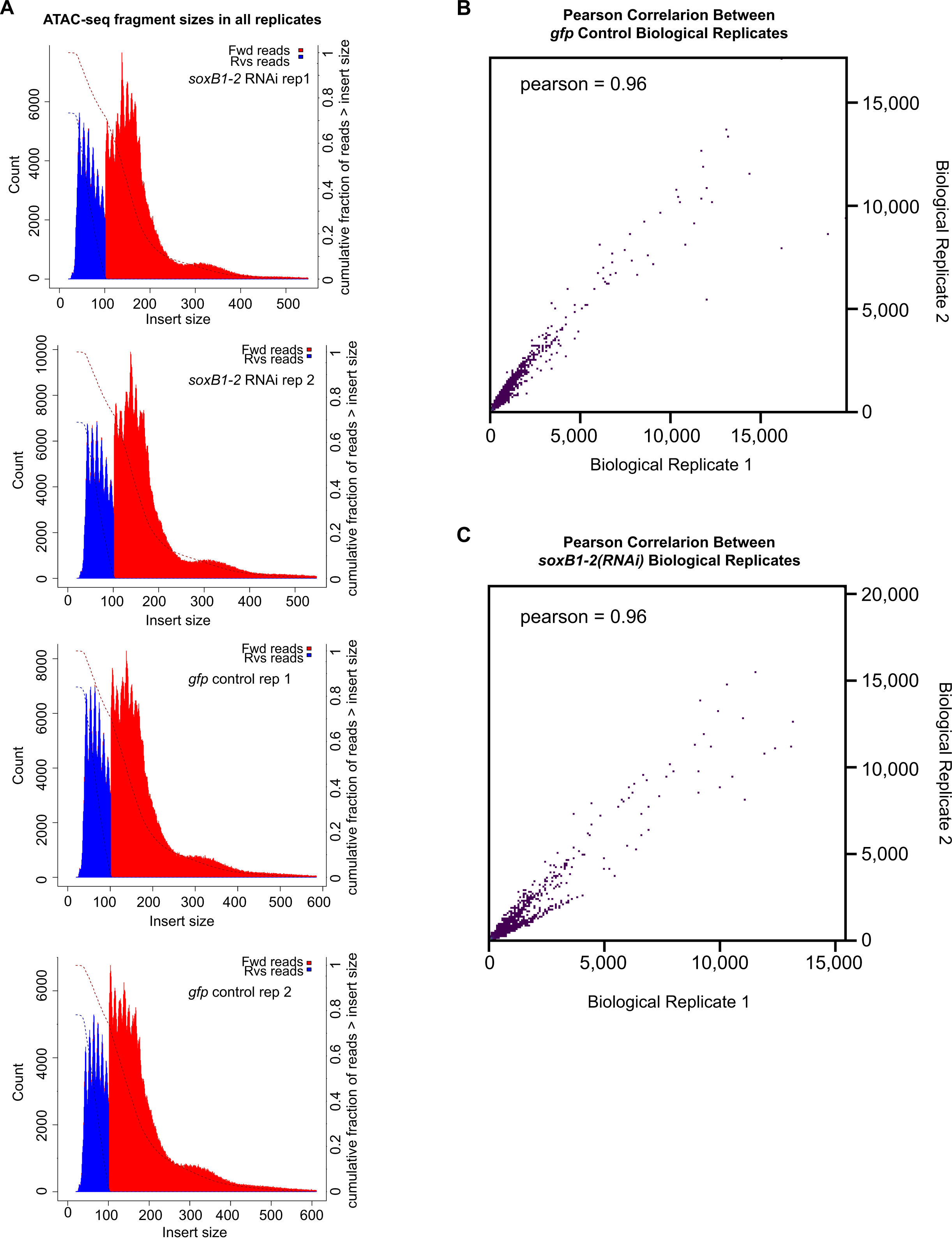

Supplement: iyag002_Supplementary_Data [file iyag002_supplementary_data.zip › Supplemental_Figure_2_GENETICS-2025-308887.tif]

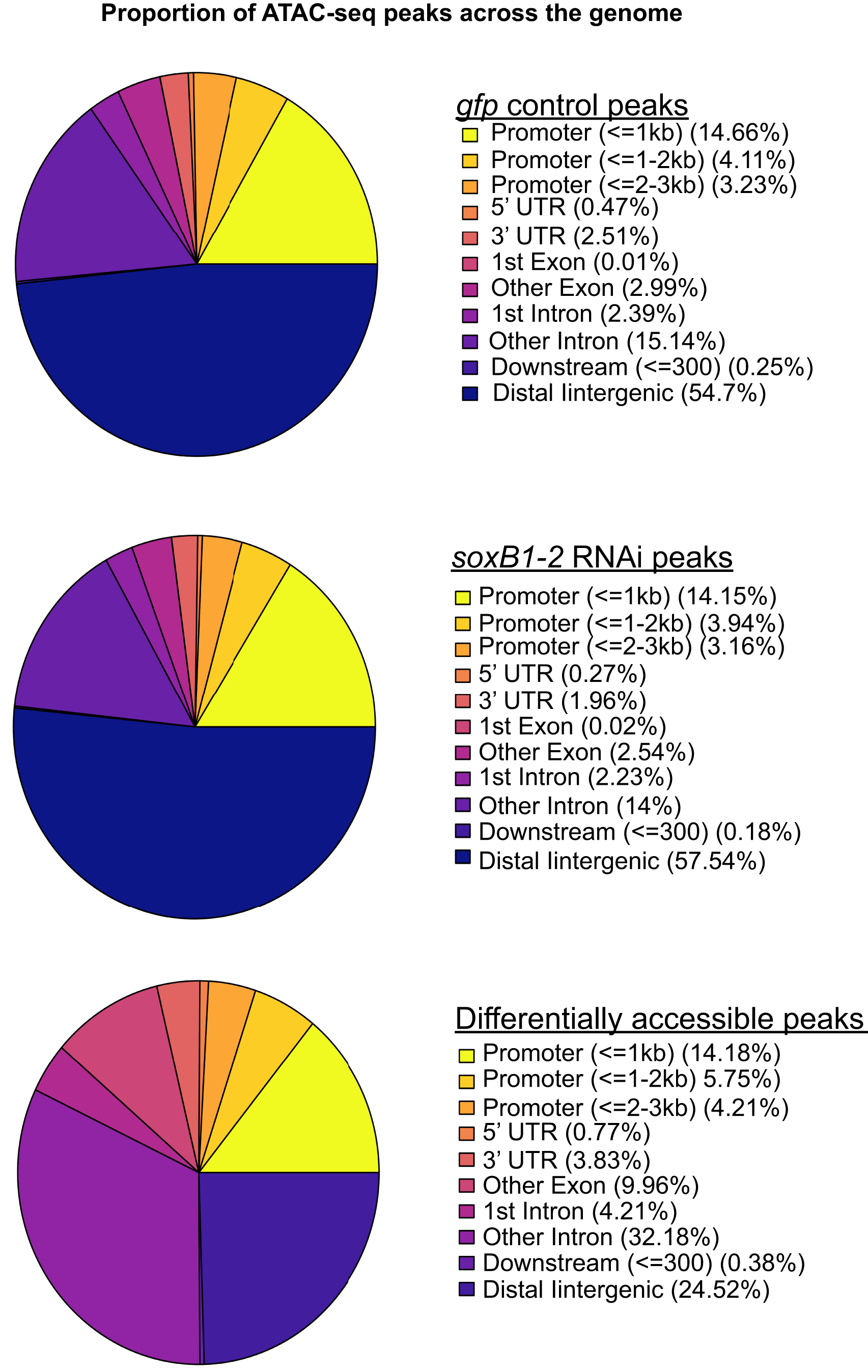

Supplement: iyag002_Supplementary_Data [file iyag002_supplementary_data.zip › Supplemental_Figure_3_GENETICS-2025-308887.tif]

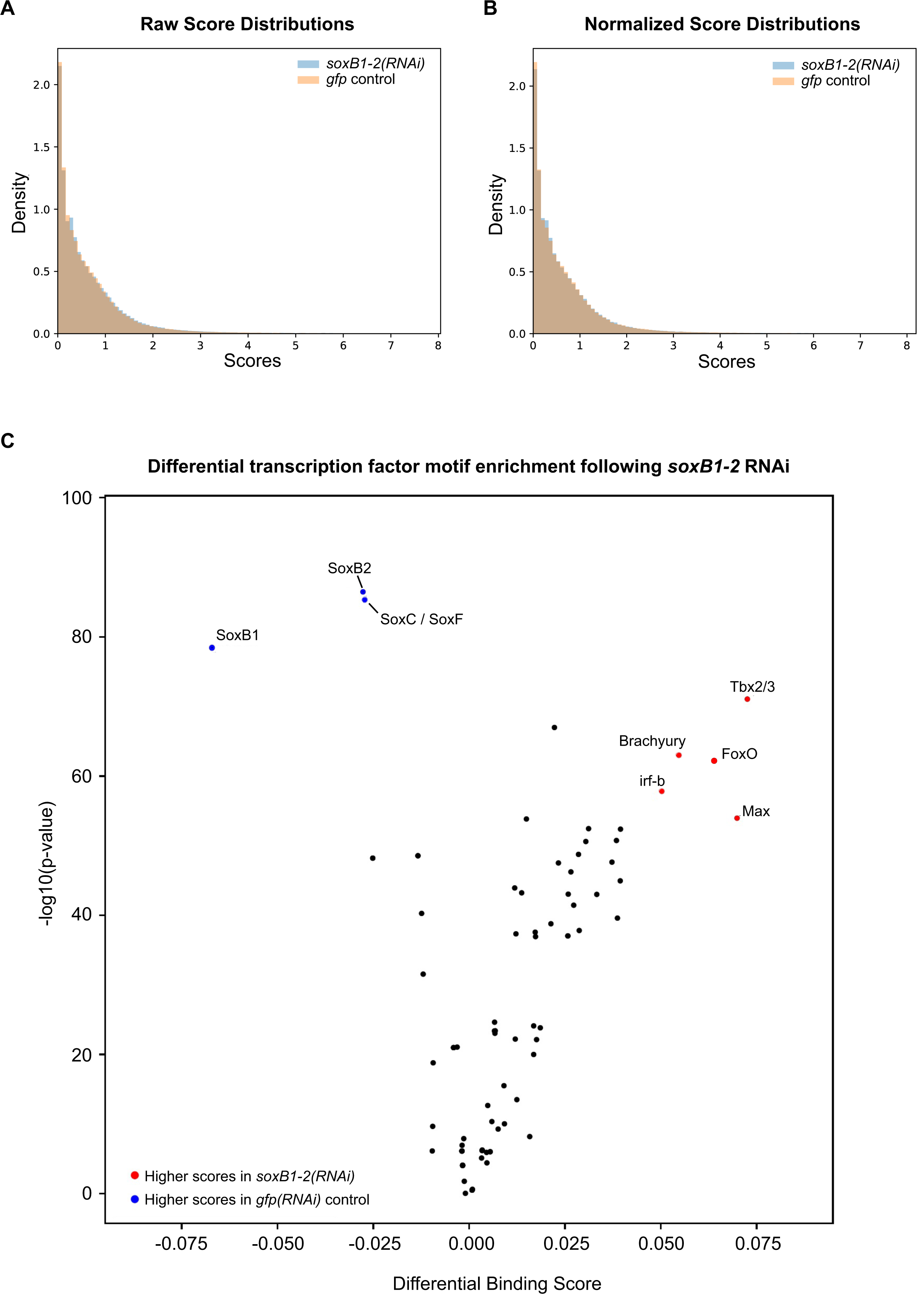

Supplement: iyag002_Supplementary_Data [file iyag002_supplementary_data.zip › Supplemental_Figure_4_GENETICS-2025-308887.tif]

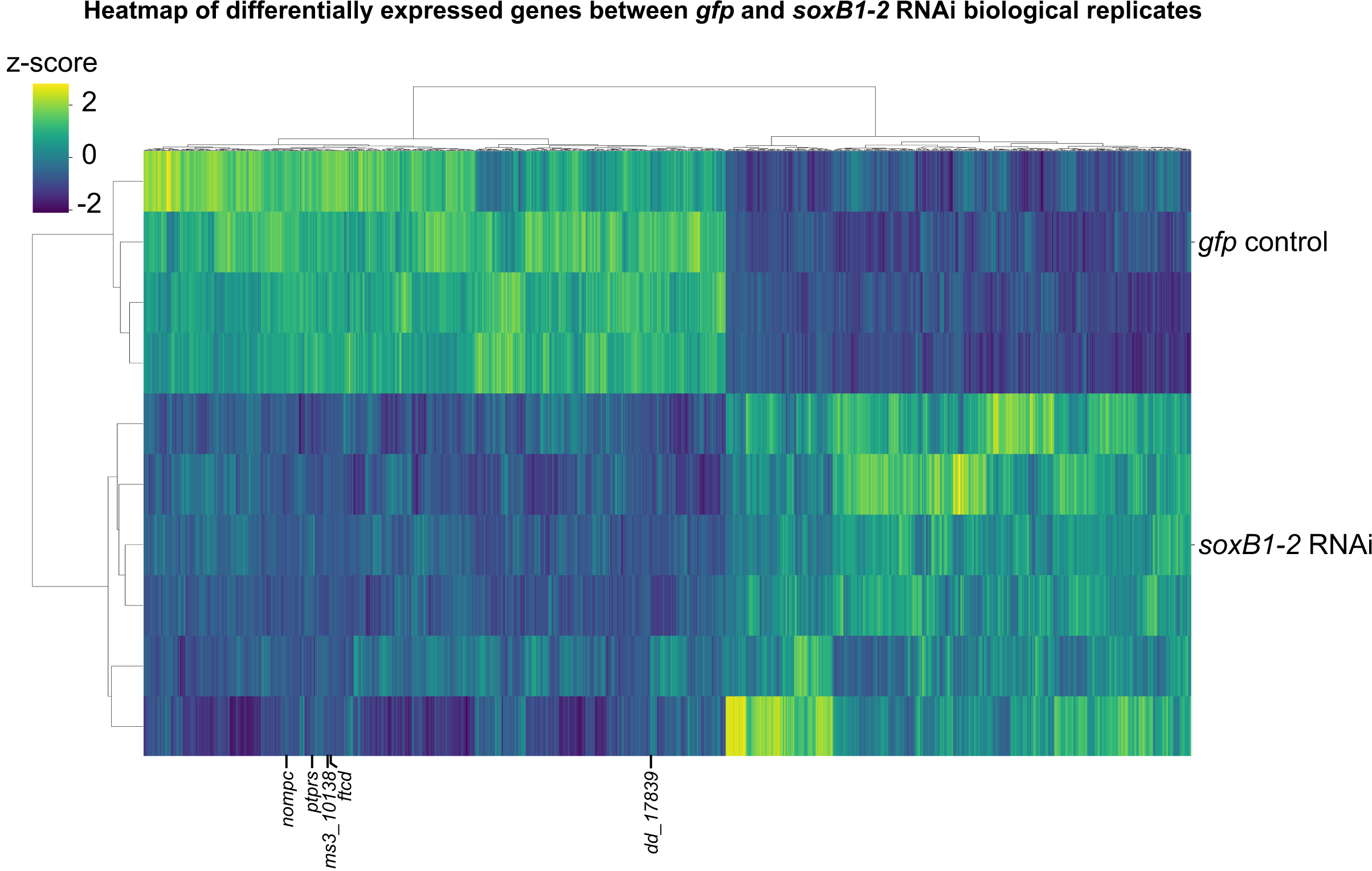

Supplement: iyag002_Supplementary_Data [file iyag002_supplementary_data.zip › Supplemental_Figure_5_GENETICS-2025-308887.tif]

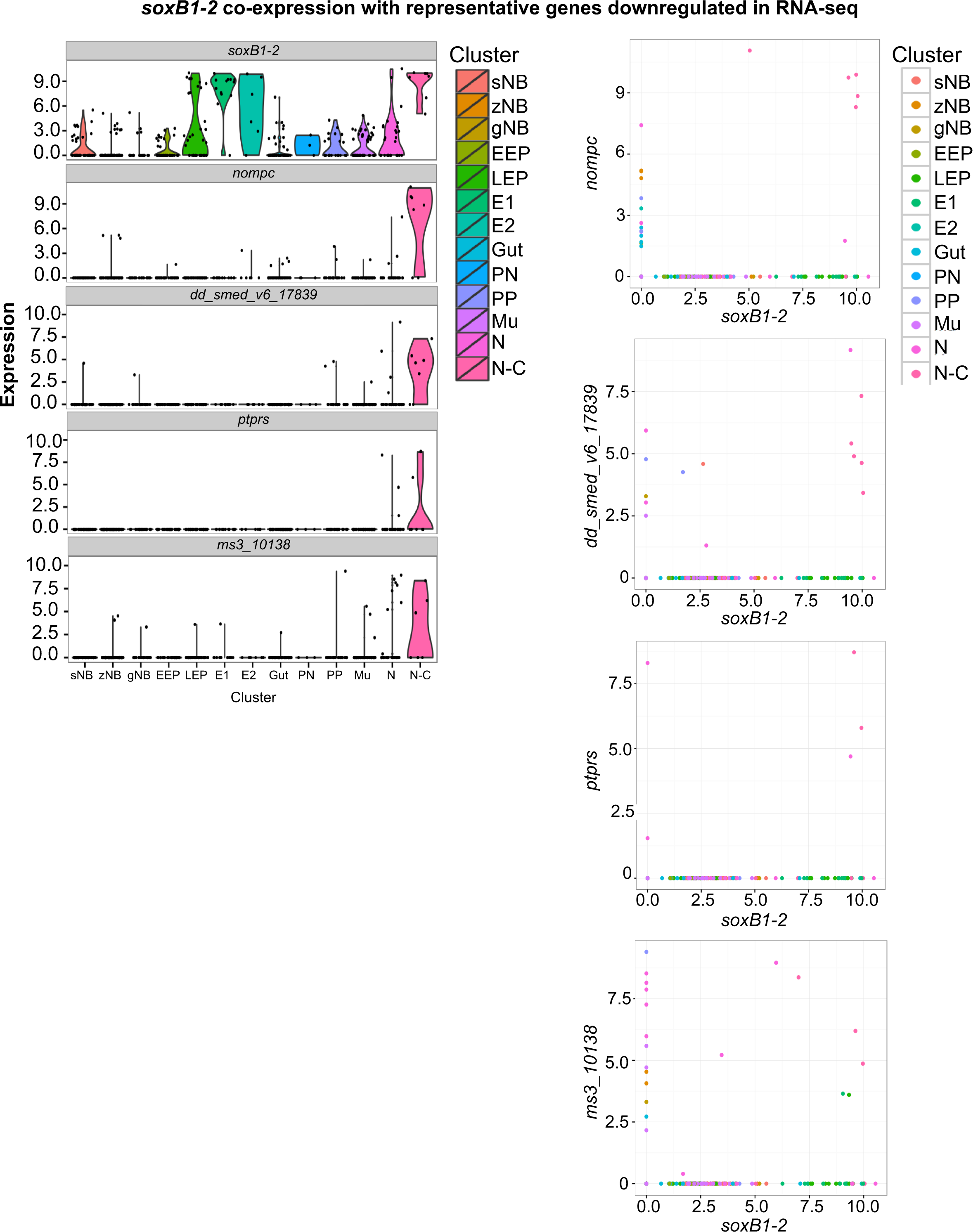

Supplement: iyag002_Supplementary_Data [file iyag002_supplementary_data.zip › Supplemental_Figure_6_GENETICS-2025-308887.tif]

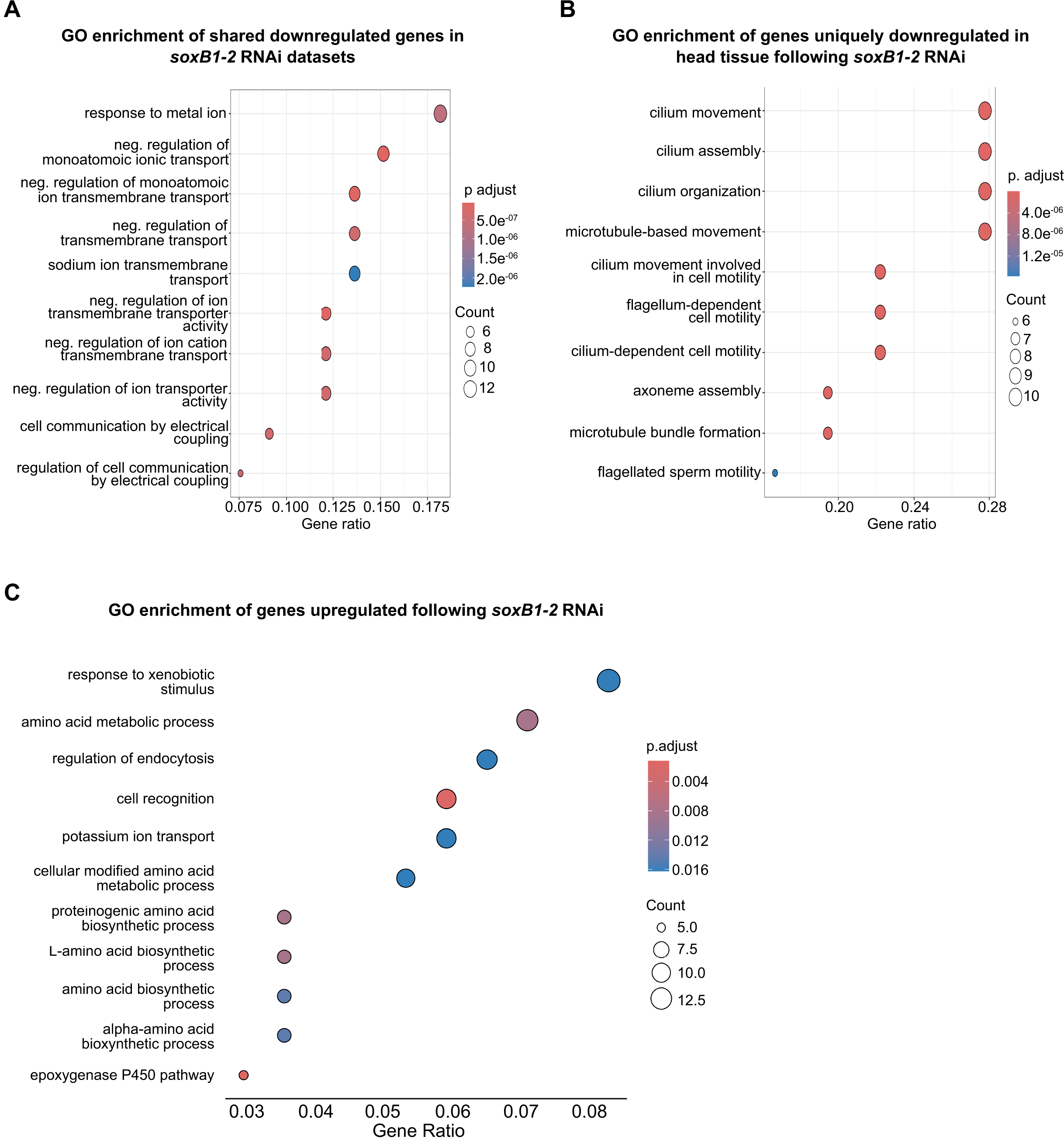

Supplement: iyag002_Supplementary_Data [file iyag002_supplementary_data.zip › Supplemental_Figure_7_GENETICS-2025-308887.tif]

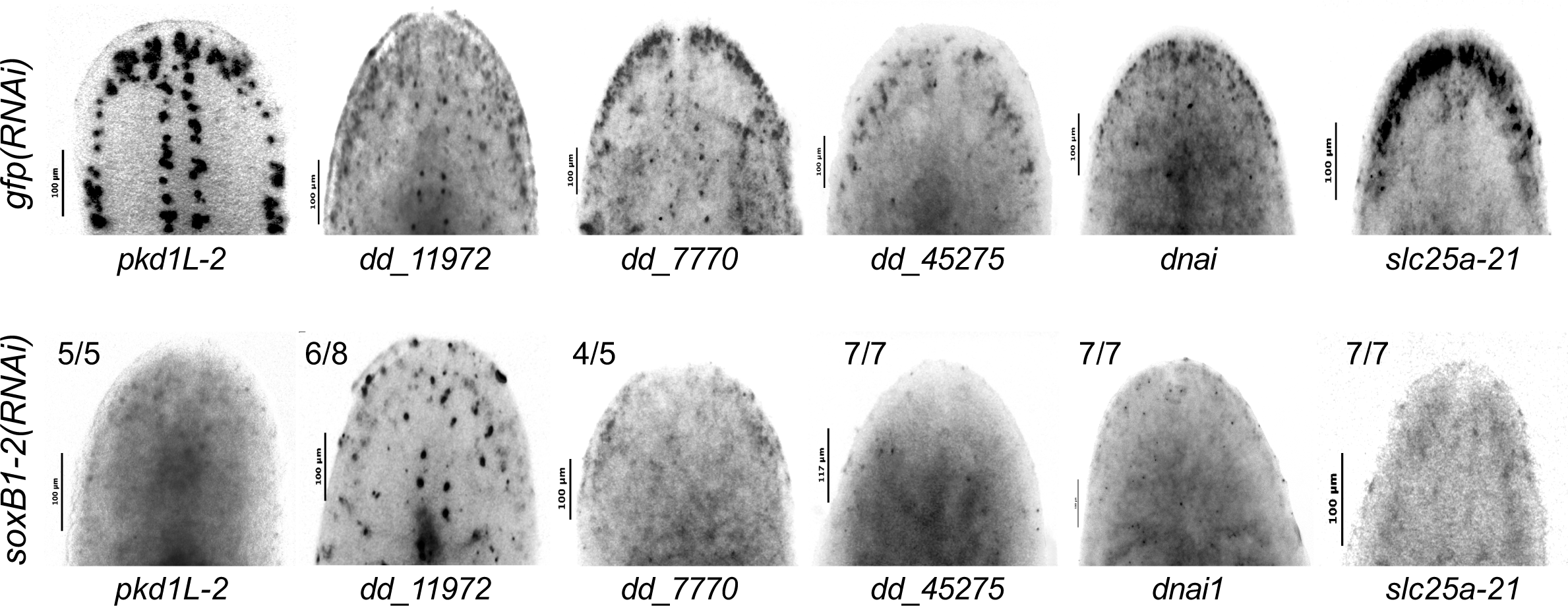

Supplement: iyag002_Supplementary_Data [file iyag002_supplementary_data.zip › Supplemental_Figure_8_GENETICS-2025-308887.tif]

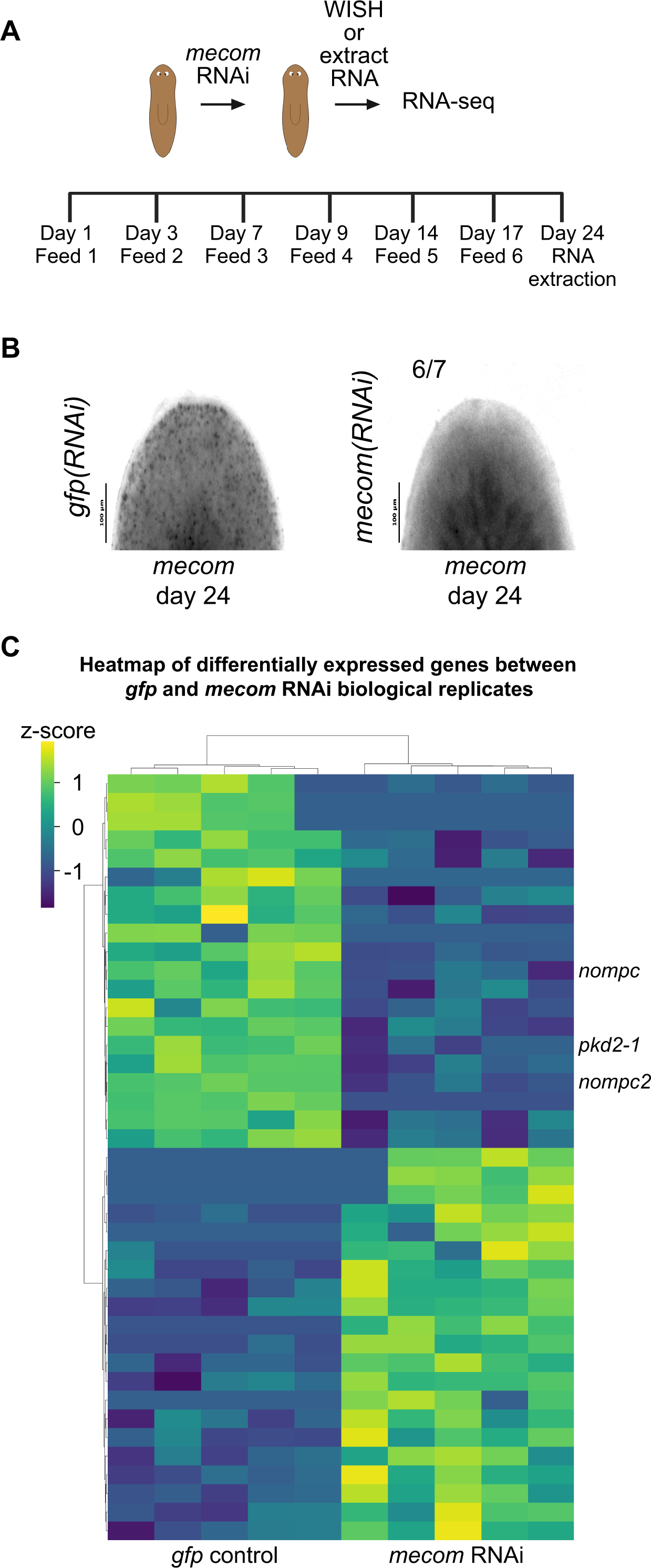

Supplement: iyag002_Supplementary_Data [file iyag002_supplementary_data.zip › Supplemental_Figure_9_GENETICS-2025-308887.tif]
